# Supplementary material for: MetaRibo-Seq measures translation in microbiomes
Source: Nat Commun. 2020 Jun 29;11:3268. doi: 10.1038/s41467-020-17081-z (PMC7324362; doi:10.1038/s41467-020-17081-z)
Supplement: Supplementary file 10 — Supplementary Data 7 [file 41467_2020_17081_MOESM10_ESM.zip › File2/Confidence_High_Taxonomy/438039_out.krona.html]

Javascript must be enabled to view this page.

members
magnitude
magnitudeUnassigned
count
unassigned
taxon
rank

438039\_out

69

superkingdom
69
2

69
phylum
976

69
class
200643

171549
order
69

815
69
family

816

SRS012849\_contig\_number\_16689SRS013476\_contig\_number\_5867SRS015960\_contig\_number\_7818SRS017103\_contig\_number\_14319SRS017191\_contig\_number\_490SRS017821\_contig\_number\_9854SRS020394\_contig\_number\_14810SRS022137\_contig\_number\_11447SRS023583\_contig\_number\_366SRS024331\_contig\_number\_11326SRS024435\_contig\_number\_44323SRS043701\_contig\_number\_2032SRS046717\_contig\_number\_2935SRS050925\_contig\_number\_33455SRS052078\_contig\_number\_2537SRS053649\_contig\_number\_985SRS055982\_contig\_number\_13601SRS056259\_contig\_number\_2429SRS063985\_contig\_number\_30793SRS064232\_contig\_number\_contig-100\_986.35120SRS064757\_contig\_number\_7514SRS1055043\_contig\_number\_contig-100\_966.155451SRS145497\_contig\_number\_3518SRS147653\_contig\_number\_contig-100\_997.56328SRS148874\_contig\_number\_2330SRS893292\_contig\_number\_3295SRS893295\_contig\_number\_contig-100\_1354.74999SRS893300\_contig\_number\_1897SRS893342\_contig\_number\_3052
genus
29
69

species
7

SRS014613\_contig\_number\_13287SRS048060\_contig\_number\_10003SRS064276\_contig\_number\_19070SRS075963\_contig\_number\_contig-100\_388.388SRS1041145\_contig\_number\_13602SRS1054691\_contig\_number\_9278SRS143598\_contig\_number\_2285
2292935


SRS1055099\_contig\_number\_11687
2292941
species
1

6
species

SRS011134\_contig\_number\_contig-100\_908.302394SRS017701\_contig\_number\_7881SRS023715\_contig\_number\_16372SRS043667\_contig\_number\_1366SRS098717\_contig\_number\_14962SRS1055067\_contig\_number\_14553
821


SRS015794\_contig\_number\_1464
338188
1
species


SRS043001\_contig\_number\_692
2292943
species
1

2292923

SRS016381\_contig\_number\_2479
1
species

47678

SRS078419\_contig\_number\_20359
species
1

species
17
357276

SRS013940\_contig\_number\_4243SRS014923\_contig\_number\_45692SRS016018\_contig\_number\_2878SRS018049\_contig\_number\_189SRS019286\_contig\_number\_2304SRS042628\_contig\_number\_14777SRS045195\_contig\_number\_1355SRS045713\_contig\_number\_9354SRS077127\_contig\_number\_16944SRS098655\_contig\_number\_3324SRS101268\_contig\_number\_1292SRS1041038\_contig\_number\_15216SRS1041039\_contig\_number\_27531SRS1041136\_contig\_number\_16607SRS1041137\_contig\_number\_12682SRS104636\_contig\_number\_14730SRS148511\_contig\_number\_8244

818

SRS018575\_contig\_number\_1765SRS056519\_contig\_number\_1505SRS101433\_contig\_number\_8813
species
3

species
1
246787

SRS024132\_contig\_number\_21519

1946083

SRS043768\_contig\_number\_contig-100\_1056.182493
species
1
